# Falls Assessment and Prevention in Older Adult Mental Health Wards

**DOI:** 10.1192/bjo.2026.11423

**Published:** 2026-06-30

**Authors:** Kate Lobo, Nicole Mathews, Emma Morgan, Kirsten Wright

**Affiliations:** NHS Borders, TD6 9BS, United Kingdom

## Abstract

**Aims:**

We aimed to evaluate the number and circumstances of falls in our 6-bed older adult ward and our 12-bed dementia specialist unit.

**Methods:**

Records were reviewed if the patient had falls ≥2 during admission. Data was collected in October and December from our records and our incident reporting system. Education sessions were held between audit cycles and medications reviewed using STOP-STARTT criteria after ward rounds.

**Results:**

From initial audit (n=12) to repeat audit (n=10) falls were approximately similar, 25 and 22 respectively. PRN use associated with the falls were 32% in October and 45% in December, and number of patients with ACB score of 3+ was 7 and 6 respectively. Physical observations taken post-fall increased from 44% to 82%, but incident reporting decreased from 84% to 36%. In the initial audit one patient accounted for 36% of the falls with 3 having not fallen, and in repeat audit 2 patients accounted for 77% of falls, with 6 having not fallen.

**Conclusion:**

All patients had received MDT input with falls bundles and care plans andregular physiotherapy review. Pre-existing extensive falls protocols are well-adhered by staff and the majority of ACB burden is due to necessary medications for stress and distress which limits ability to reduce falls risk. We identified there is ongoing scope for medication reconciliation, and there is a follow-up QIP starting to evaluate de-prescribing medication in this high-risk cohort with geriatrician advice.

